# Mechanical intelligence for learning embodied sensor-object relationships

**DOI:** 10.1038/s41467-022-31795-2

**Published:** 2022-07-15

**Authors:** Ahalya Prabhakar, Todd Murphey

**Affiliations:** 1 École Polytechnique Fédérale de Lausanne (EPFL), Lausanne, Switzerland; 2 Northwestern University, Chicago, IL USA

**Keywords:** Mechanical engineering, Computer science

## Abstract

Intelligence involves processing sensory experiences into representations useful for prediction. Understanding sensory experiences and building these contextual representations without prior knowledge of sensor models and environment is a challenging unsupervised learning problem. Current machine learning methods process new sensory data using prior knowledge defined by either domain knowledge or datasets. When datasets are not available, data acquisition is needed, though automating exploration in support of learning is still an unsolved problem. Here we develop a method that enables agents to efficiently collect data for learning a predictive sensor model—without requiring domain knowledge, human input, or previously existing data—using ergodicity to specify the data acquisition process. This approach is based entirely on data-driven sensor characteristics rather than predefined knowledge of the sensor model and its physical characteristics. We learn higher quality models with lower energy expenditure during exploration for data acquisition compared to competing approaches, including both random sampling and information maximization. In addition to applications in autonomy, our approach provides a potential model of how animals use their motor control to develop high quality models of their sensors (sight, sound, touch) before having knowledge of their sensor capabilities or their surrounding environment.

## Abstract (web summary)

Information-based search strategies are relevant for the learning of interacting agents dynamics and usually need predefined data. The authors propose a method to collect data for learning a predictive sensor model, without requiring domain knowledge, human input, or previously existing data.

## Introduction

A defining characteristic of intelligence and learning is the ability to process sensory experiences into concise representations from which one can predict and anticipate sensory experience at new states^1–3^. Humans recognize and encode complex scenes and objects in memory with remarkable accuracy, able to imagine them from different viewpoints or places^1–3^. Recent efforts have focused on developing neural networks and models for learning concise representations from sensory data^4,5^, but these approaches require that datasets for learning already exist. The challenge of data acquisition in support of learning, when these datasets are not available, remains unsolved. This lack of a theory explaining where data should come from affects all data-driven science^6^ since the quality of machine intelligence and resulting human behavior depends on quality of data. Information-based search strategies have been used for both modeling animal behavior^7,8^ and synthesizing behavior^9^ for artificial systems. However, there are no principles explaining how mechanical motion supports general models of learning. This paper creates a general model of how agents use motor capability to support learning when the data for learning needs to be physically acquired—a typically necessary step that is often not considered in the machine learning literature that uses canned datasets acquired elsewhere. When training data acquired for learning is dependent on exploratory strategies throughout the environment, principles for motion control become a cornerstone of principles for learning.

In this paper, we use the challenge of learning predictive sensor models without predefined knowledge of the sensor or environment as a setting for deriving physics-constrained motion strategies for learning. Our method works well without modification for both near-field and far-field sensor examples. We accomplish this using a motor principle based on ergodicity—a physically important property from statistical mechanics^10^ that relates temporal behavior of trajectories to spatial distributions—with respect to information-rich regions of a physical domain. This approach results in dramatically higher quality learned models with lower energy expenditure than approaches that rely on randomized sampling of the environment, the dominant data acquisition strategy in nearly all machine learning. Moreover, the approach presented here outperforms entropy-based information maximization in terms of learning performance (see Supplementary Information).

Our learning framework is based on autoencoders; these learn an encoder-decoder framework that encodes input data into a lower-dimensional latent representation from which signals are decoded for prediction^11^. Conditional autoencoders learn a predictive model by learning a latent space conditioned on external factors (e.g., agent pose). Specifically, we learn a sensory model of the form (${y}^{\prime}=f({\theta }_{e},{\theta }_{d},{x}^{\prime})$)—where *θ*_*e*_ and *θ*_*d*_ parameterize an encoder and decoder in an autoencoder that has a latent space *z* determined from a seed observation at robot state *x*_0_—in order to predict what the agent would experience at a new state ${x}^{\prime}$. Active data acquisition is specified by minimizing the distance from ergodicity^12^ between the time-averaged statistics of an agent along a trajectory and a target coverage spatial distribution (that represents how information about the autoencoder is distributed spatially). Ergodicity with respect to the entropy of variables in a learning representation is a principle of motion, much like energy minimization^13,14^ and entropy^7^.

### Metrics on ergodicity for synthesizing motion for active learning

Unsupervised learning for predictive sensory models (e.g., scene prediction using data from sensors in self-driving cars) relies on processing and learning from batches of data received continuously and analyzed intermittently. This episodic framework benefits from a data acquisition approach that is simultaneously responsive to changes in the learning representation (e.g., changes to a deep neural network) and persistent in its data collection while waiting for those changes. Unlike other prediction-based reward functions that focus on acquisition of unusual measurements^15^, metrics on ergodicity—measures that indicate how well a particular trajectory *x*(*t*) covers a specified distribution *ϕ*(*x*)—enable the formulation of a controlled response to the need for data that seeks out informative regions based on the entropy of the learned model over the exploration space, generating mechanical motion that actively aids intelligent learning. Ergodic control—based on a metric on ergodicity—enables synthesis of exploratory motions that adapt in response to data collected and evolving information distributions.

**Fig. 1:**
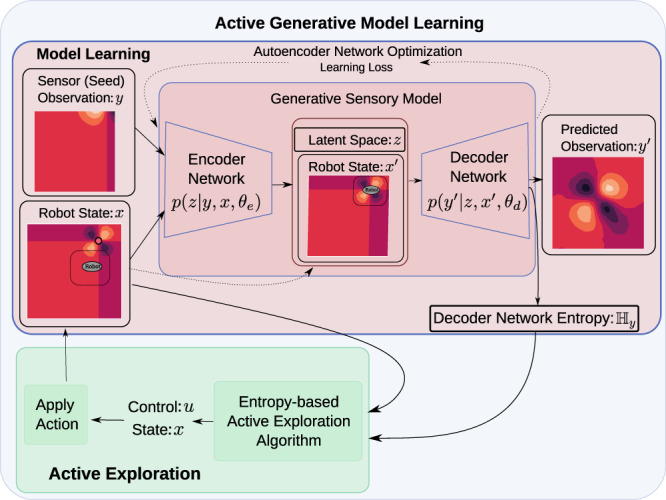
Model learning approach. The learning approach consists of a learning algorithm for creating generative sensory models from data and an active exploration framework that collects data based on the conditional entropy of the learned model. Conditional autoencoders are used to learn a low-dimensional latent encoding of the high-dimensional sensor input. Sensor output at novel states—the generative aspect of the model—is then predicted using this low-dimensional encoding. We develop an active exploration approach for data acquisition based on ergodic coverage of the entropy of the latent space to improve model uncertainty. This framework demonstrates a feasible approach for real-time, generalizable sensor model learning without the need for predefined domain knowledge of the environment or sensor structure.

Autoencoders provide an estimate of a latent space variable *z*, explicitly dependent on a conditional variable *x*, based on a sensor observation *y*. As learning progresses, the parameters of the encoder and decoder networks—*θ*_*e*_ and *θ*_*d*_, respectively—are optimized to interpret input data to best predict sensory data, such that it outputs both a prediction of the sensor observation ${y}^{\prime}$ and its entropy ${{\mathbb{H}}}_{y}$. As illustrated in Fig. 1, the state of a sensor is the conditional variable in this framework; the learned representation changes as the sensor state changes (e.g., pose of a camera or pressure and location of a tactile sensor). Conditional autoencoders are the representation of learning we focus on here, but their key characteristic is that the learning representation is explicitly conditioned on something over which an agent has control; the arguments used here in the context of conditional autoencoders would apply to other learning methodologies with this same property.

Since the entropy estimate approximates how information in the autoencoder depends on the state of the sensor, we maximize the ergodicity of the sensor trajectory with respect to entropy of the autoencoder model to dictate how the sensor moves in order to acquire more information about the unknown or partially known autoencoder. We assume that the conditional variable *x* is a state of a controllable system dependent on an input *u*, and that the dynamics $\dot{x}=f(x,u)$ of the sensor are known (see Supplementary Information). We numerically optimize *u* to be maximally ergodic with respect to ${{\mathbb{H}}}_{y}$, the entropy of the decoder network by minimizing a metric on ergodicity, where in this case *ϕ*(*x*) is ${{\mathbb{H}}}_{y}$ conditioned on *x* and the latent space representation *z* derived from the current *x*, *y* (also discussed in the Supplementary Information). We refer to this control as *ergodic sampling*.

## Results

Near-field sensors (e.g., touch, electrosense) and far-field sensors (e.g., vision, hearing) depend differently on actions taken by an agent. In the case of vision, actions that support learning involve getting a target within the field of view at a sufficient distance to identify visual features, avoiding motion to avoid blur. In contrast, touch requires proximity to a target and often requires motion relative to the target; information content drops dramatically as soon as a sensor is out of contact. The ergodic sampling method applies to both near-field and far-field sensory learning without modification. We demonstrate this using electrosense physics as a near-field sensory model and vision as a far-field model.

**Fig. 2:**
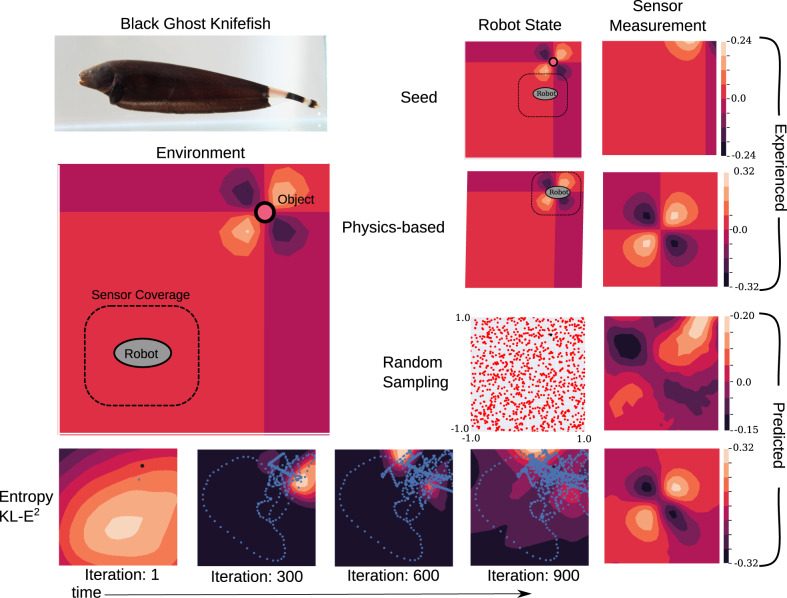
Electrosense model learning. We compare the predicted measurement of the learned electrosensory model to the physics-based electrosensory model. The model is an approximation of the sensory system of a weakly electric fish (the black ghost knifefish^16^). Using a seed image, we show the reconstruction of the physics-based measurement field estimate near the object interest, using a conditional autoencoder with active learning using random sampling, and the proposed ergodic sampling strategy. The proposed approach explores the environment based on the evolution of the entropy of the latent space over time (shown evolving over time along with the resulting trajectory shown in blue). Despite no a priori information about the object's location (indicated with black markers) or measurement model, as time evolves the entropy of the latent space—representing where the information affecting the model estimate is highest—converges near the object location and the ergodic sampling spends significantly more time exploring that region without fixating on a particular state. As a result, the ergodic sampling approach results in a significantly better reconstruction of the predicted sensor observation with much lower energy expenditure needed during exploration.

First, we explore the benefits of ergodic sampling for learning an electrosensory model. The electrosensory modality—commonly referred to as electrolocation—is used by a group of South American weakly electric freshwater nocturnal fish that hunts in darkly tinted waters where visibility is low. Understanding their unique sensing modality and decision-making has been of interest both for understanding biological behavior and for using these sensor modalities in robotics^16–18^. Electrosense is a near-field sensory modality that only produces electrical perturbations in voltage observations when near the object^16^, highlighting the importance of active data acquisition—like touch, model generation is impossible without movement. In the case of weakly electric fish, electrosense measurements are distributed across the surface of the fish body, yielding a sensor where each voltage measurement can be thought of as a pixel in an image. However, these sensory activity readings are proportional to the voltage difference between inside and outside the skin at the sensor, so the geometry of the pixels does not directly correspond to the geometry of the environment, the way a camera’s pixels do. Similar to a camera image, the signal space of the electrosensory modality is high dimensional. We construct an electrosense measurement model (described in the Supplementary Information) of an underwater conductor in an environment with a spherical object at an unknown location. In Fig. 2 we compare the results of electrosensory model learning using ergodic sampling and compare to random sampling as a benchmark—reflecting the ideal sampling around the workspace without having to move according to the laws of physics (see Supplementary Information for details of numerical experiments).

Trajectories spend time close to the object, as seen in Fig. 2 (bottom). Without any prior knowledge of the sensor measurement model or the object, this method automatically detects key aspects of near-field sensing without those features being pre-specified. The sensor model has a 441-dimensional sensor observation *y*—which consist of voltage differentials evenly distributed in a 21 × 21 grid (covering the sensor coverage region indicated in Fig. 2) over the center of the robot—and the robot state *x* and encodes *y* into a 2-dimensional latent space representation *z* conditioned on *x*. The latent space representation was determined from a single seed observation (top right) with the agent sufficiently near the object to register an electrical perturbation—an observation sufficient for determining features of perturbations but insufficient to otherwise describe the location of the conductor (other details are provided in the Supplementary Information).

The sensor model learned using the ergodicity-based dataset results in a better reconstruction of the analysis-based measurement model (right, second from top) than the sensor model learned with randomly sampled data (right, second from bottom). Moreover, the data acquisition technique spends time in locations that cover regions of high information about voltage perturbations (bottom), so that the resulting predicted sensor output (bottom right) accurately captures the object’s location and electrical signals experienced at a novel agent state.

**Fig. 3:**
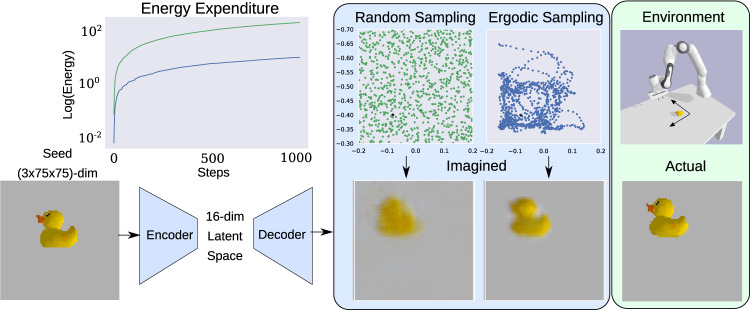
RGB camera model learning. We compare the predicted measurement generated from the learned 3-channel RGB camera model. Using a seed image, we show the reconstruction of the visual camera field estimate near the object interest, using a CVAE with active learning using random sampling, and the proposed ergodic sampling strategy. Despite no a priori information about the object's location or measurement model, as time evolves, ergodic sampling spends significantly more time exploring the region around the object in the environment. However, unlike the near-field electrosensory model, the camera characteristics generates exploration further away from the object, where the object appears in the periphery of the camera view, instead of remaining directly over the object. The ergodic sampling approach (shown in blue) requires much lower energy expenditure compared to random sampling (shown in green). Furthermore, the ergodic sampling approach results in a significantly better reconstruction of the predicted sensor observation, better capturing the object's characteristics (i.e, shape, color) and the characteristics of the sensor-environment interactions (i.e., camera lighting). More detail can be found in the Supplementary Information.

We next consider the learning problem for high-dimensional vision sensors in environments with complex, nonparameterizable objects (e.g., objects with arbitrary shapes and visual textures) and multiple regions of interest (e.g., when there are multiple objects present). We construct a vision-based model of a three dimensional object for a 3-channel 75 × 75 pixel RGB camera attached to a robot arm in a simulated Pybullet^19^ environment as shown in Fig. 3. The 75 × 75 × 3 = 16,875-dimensional sensor output uses a CNN-CVAE (from ref. ^20^ and described in the Supplementary Information) for learning and encoding a 16-dimensional latent space representation. The simulated environment consists of a tabletop with a rubber duck on it—an object with complicated shapes and textures.

The random sampling generates samples over the entire workspace, while the ergodic sampling controller spends more time acquiring data near—but not on top of—the object in the environment, where the highest-value information in the environment is distributed (Fig. 3 and Supplementary Movie 1). Unlike the near-field electrosense example, where the robot needed to be very close to the object to acquire informative data, the ergodic sampling algorithm samples further away from the object for vision modeling. Exploration is not guided by any domain knowledge—the exploration behavior arises entirely based on the entropy of the unknown sensory model during the learning process and the maximally ergodic response with respect to that entropy.

The learned vision model of the object predicts the resulting camera image at every agent location within the domain, many of which were not in the training dataset generated during the data acquisition process. With ergodic sampling the learned model generates a higher quality reconstruction of the object in the camera image than the one generated from random sampling, reflecting the object’s location accurately and capturing the complex shape and lighting of the object in the RGB camera model. Furthermore, the energy used to explore the environment and acquire data is lower over time for ergodic sampling than uniform random sampling, indicating that one can both improve energetic efficiency while also improving learning by collecting high-quality data.

Lastly, an example of modeling multiple objects using a simulated black and white camera is treated in detail in the Supplementary Information. There we see two important features of ergodic sampling with respect to the entropy of the learned model—that the exploratory trajectories inspect each object individually, without requiring a representation of the objects or their number, and that the resulting predictive model is able to discriminate between the objects based on the learning updates when random sampling-based learning cannot. Moreover, we use this example to illustrate the advantages of ergodic sampling over entropy-based information maximization. In the case of information maximization, the active learning strategy fixates on just one object, whereas the ergodic sampling strategy collects information about all the features about both objects, leading to substantially lower testing loss.

## Discussion

The use of learning-for-control dominates much of modern robotics and has its roots in reinforcement learning^21^. Here, we develop a theory of control-for-learning, based on a principle of maximal ergodicity with respect to entropy of a learning algorithm.

Sensor-environment model learning often relies on domain knowledge^22–26^ or is hand tailored to specific domains^27–29^. Other unsupervised model learning approaches assume predefined knowledge of sensor structure^30^ or assume large pre-existing datasets^4,5^. Oftentimes these environments are simulation-based—permitting sampling the state space without adhering to the laws of physics or even continuity of the state in time—so they do not examine the data acquisition process or optimization of the learning representation. Other work examines the quality of sensor data, but does so independently from the learning representation. For instance, Bayesian surprise in sensory data is used to identify data needed to guide human behavior^31–33^ and to generate classifiers and event detection in sensors and robots^34–36^, but these techniques focused on analyzing the value of existing data rather than guiding exploration and data acquisition. Such methods do not aim to provide approaches to actively acquire informative data in the physical world subject to physical constraints. Even reinforcement learning techniques treat data acquisition as an afterthought (discussed in the Supplementary Information).

This work proposes a domain-independent framework that integrates data acquisition and learning processes using ergodicity—familiar to most as a measure from statistical mechanics. We demonstrate that an ergodicity-based approach enables active acquisition of informative sensor data to efficiently learn a sensor model. The approach is amenable to high-dimensional sensor outputs and complex learning models in a variety of scenes and environments; here we demonstrate with both near-field and far-field sensor types with different physical properties. The ergodic sampling algorithm capable of exploring high-dimensional search spaces in real-time was developed in ref. ^37^. Here, we further developed it for exploring with respect to entropy of learning models. Critically, we show how ergodicity with respect to the state of the neural network can be used to actively acquire informative data within an unsupervised learning framework, enabling active data acquisition that evolves along with the unsupervised model learning. The ergodic framework enables exploration without requiring physics-based knowledge of a sensor or the environment (but does assume ability to move the sensor). This work provides a framework for all sensor-environment learning that does not rely on physics-based models to learn and predict experiences, but rather uses experienced observations along with the ability to move in an environment to guide and inform model learning and future behavior.

Although this paper has focused on synthetic forms of mechanical motion that approximate ergodicity as a principle for learning, mechanical systems that are automatically ergodic as a result of their statistical mechanical properties would learn without needing this synthesis. This indicates how learning may be a consequence of mechanical coverage combined with computing updates, in some cases making learning as much a mechanical property^38^ as an algorithmic property of a system.

## Methods

In the following section, we describe the methods for learning a generative sensory model with no a priori information, human input, or sensor domain knowledge. We describe a Kullback–Leibler (KL)-ergodic measure, a sample-based approximation of the ergodic metric, and an optimal controller to enable ergodic exploration that extends to high-dimensional exploration spaces. Using this, we present an exploration strategy for acquiring data from regions of high information for efficient learning.

### Assumptions

Assume we have an agent with system dynamics that can be modeled as

1 $$\dot{x}(t)=f(x(t),u(t))$$

2 $$=g(x(t))+h(x(t))u(t)$$

where $x\in X\subset {{\mathbb{R}}}^{n}$ is the state of the agent, $u\in {{{{{{{\mathcal{U}}}}}}}}\subset {{\mathbb{R}}}^{m}$ is a control vector, *f*(*x*, *u*) is the (possibly nonlinear) transition model, which we split into $g(x)\subset {{\mathbb{R}}}^{n}\to {{\mathbb{R}}}^{n}$, the free (unactuated) dynamics, and $h(x):{{\mathbb{R}}}^{n}\to {{\mathbb{R}}}^{n\times m}$, the input vector fields. It has a sensor that outputs data $y\in Y\subset {{\mathbb{R}}}^{o}$, which here comes from simulated sensors.

The agent explores a workspace ${{{{{{{\mathcal{X}}}}}}}}$ with no a priori knowledge of environmental physical characteristics (e.g. objects in the environment or textures).

### Model learning

We use a conditional variational autoencoder (CVAE) to construct a generative sensor model of the sensing modality. We define the inputs to the CVAE encoder as the sensor data *y*(*t*) and agent state *x*(*t*). We model both the encoder and decoder as neural networks. The encoder network is a probability distribution ${p}_{{\theta }_{e}}(z| y,x)$ parameterized by *θ*_*e*_ that compresses the high-dimensional sensor measurement into a low-dimensional compressed latent space *z*. The decoder network is a probability distribution ${p}_{{\theta }_{d}}(y| z,x)$ parameterized by *θ*_*d*_ that has as its input *z*_*x*_—generated by sampling the normally-distributed latent space *z* such that *z*_*x*_ = *z*_*μ*_ + *z*_*σ*_*ϵ*, where *ϵ* ~ Normal(0, 1) and the agent state *x*(*t*). The outputs from the decoder are the expected sensor readings $\tilde{y}(t)$ conditioned on the agent state *x*(*t*).

#### Electrosense

The electrosense network’s input is the 441-dimensional sensor output *y*(*t*) and the 2-dimensional agent state *x*(*t*) = [*p*_*x*_, *p*_*y*_], where *p*_*x*_, *p*_*y*_ are the positions in space at time *t*. The encoder network compresses the input with a series of linear transformation and ReLU layers into a 2-dimensional latent space *z* and its log-variance $\log [{\mathbb{V}}(z| y,x)]$. The decoder network takes in samples of the reparametrized *z* latent space and the agent state *x* and generates a prediction of the 441-dimensional measurement $\tilde{{y}_{n}}$ and a scalar value representing the log-variance $\log [{\mathbb{V}}(y| z,x)]$ representing the uncertainty with a series of linear transformation and ReLU layers.

#### Vision

 Intensity camera model: The camera model network’s input is a 1-channel 38x38 intensity image (1444-dimensional) sensor output *y*(*t*) and the 2-dimensional agent state *x*(*t*) = [*p*_*x*_, *p*_*y*_]. The encoder network compresses the input with a series of linear transformation and ReLU layers into a 16-dimensional latent space *z* and its log-variance $\log [{\mathbb{V}}(z| y,x)]$. The decoder network takes in samples of the reparametrized *z* latent space and the agent state *x* and generates a prediction of the 1444-dimensional image measurement $\tilde{y(t)}$ and its scalar log-variance $\log [{\mathbb{V}}(y| z,x)]$ with a series of linear transformation and ReLU layers.

##### RGB camera model

The CVAE for RGB camera model learning consists of a combination of convolutional neural networks and a conditional autoencoder networks (CNN-CVAE). For the encoder network, the 3-channel 75 × 75 image is first an input into a convolutional neural network (CNN) consisting of 2d convolutional layers and max pooling layers to condense the image into a lower-dimensional feature space. The flattened output of the CNN and the 2-dimensional agent state *x* = [*p*_*x*_, *p*_*y*_] is then an input into a second neural network that compresses the input with a series of linear transformation and ReLU layers into a 16-dimensional latent space *z* and its log-variance $\log [{\mathbb{V}}(z| y,x)]$. The decoder network consists of two different decoder networks, an image-decoder network and variance-decoder network, that both take as input samples of the reparametrized *z* latent space and the agent state *x*. The image-decoder network generates a prediction of the image by first using a neural network with a series of linear transformation and ReLU layers and then a deconvolutional neural network to generate the 3-channel image prediction. The variance-decoder network takes in the samples of the reparametrized *z* latent space and the agent state *x* with a series of linear transformation and ReLU layers generates a scalar value representing the log-variance, or the uncertainty, of the predicted image.

Our objective is to develop a method that is general to any form of sensor model learning that requires an agent to dynamically move to seek out measurements through actions. In the next section, we describe a KL-ergodic measure and the derived optimal controller (KL-E^2^) that specifies the active exploration task as an area-coverage objective, developed in^37,39^.

### Kullback–Leibler (KL)-ergodic measure

We define active exploration for informative data acquisition by generating actions that actively seek out informative data. The expected informativeness of a measurement at a particular state is defined to be the entropy of the learning model at that state. This is accomplished by specifying the active data acquisition task using an area coverage objective where we minimize the KL-divergence between the time average statistics of the agent along a trajectory and a spatial distribution defining the current coverage requirement. As the exact time-averaged statistics, as described in ref. ^9^, are a collection of delta functions parameterized by time, we define an approximation of the spatial statistics of the agent. To do so, we approximate the delta function as a Gaussian distribution with covariance Σ, converging to the delta function as ∥Σ∥ → 0. We define the approximation to the spatial statistics of the agent as follows:

**Definition 1** Given a search domain ${{{{{{{{\mathcal{X}}}}}}}}}^{v}\subset {{\mathbb{R}}}^{n+m}$ where *v* ≤ *n* + *m*, the Σ-approximated time-averaged statistics of the agent, i.e., the time the agent spends in regions of the search domain ${{{{{{{{\mathcal{X}}}}}}}}}^{v}$, is defined by

3 $$q(s| x(t))=\frac{1}{{T}_{r}}\int\nolimits_{{t}_{i}-{t}_{r}}^{{t}_{i}+T}\mu (s\parallel \bar{x}(t))dt$$

4 $$=\frac{1}{{T}_{r}}\int\nolimits_{{t}_{i}-{t}_{r}}^{{t}_{i}+T}\frac{1}{\eta }\exp \left[-\frac{1}{2}\parallel \left(s\right.-\bar{x}(t){\parallel }_{{{{\Sigma }}}^{-1}}^{2}\right]dt$$

where $s\in {{{{{{{{\mathcal{X}}}}}}}}}^{v}\subset {{\mathbb{R}}}^{n+m}$ is a point in the search domain ${{{{{{{{\mathcal{X}}}}}}}}}^{v}$, $\bar{x}(t)$ is the component of the agent’s state *x*(*t*) that intersects the search domain ${{{{{{{{\mathcal{X}}}}}}}}}^{v}$, ${{\Sigma }}\in {{\mathbb{R}}}^{v\times v}$ is a positive definite matrix that specifies the width of the Gaussian, *η* is a normalization factor, *t*_*i*_ is the *i*^th^ sampling time, and *T*_*r*_ = *T* + *t*_*r*_ is sum of the time horizon *T* and amount of time *t*_*r*_ the agent remembers *x*_*v*_(*t*) into the past.

Using this approximation, we are able to approximate the ergodic area-coverage objective in^9^ using the following KL-divergence objective^40^:

5 $${D}_{{{{{{{{\rm{KL}}}}}}}}}(p\parallel q)={\int}_{{{{{{{{{\mathcal{X}}}}}}}}}^{v}}p(s)\ln \frac{p(s)}{q(s)}ds$$

6 $$=\underbrace{{\int}_{{{{{{\mathcal{X}}}}}}^{v}}p(s)\ln p(s)ds}_{{{{{{{{\mathrm{does}}}}}}}}\,{{{{{{{\rm{not}}}}}}}}\,{{{{{{{\rm{depend}}}}}}}}\,{{{{{{{\rm{on}}}}}}}}\,x(t)}-\underbrace{{\int}_{{{{{{\mathcal{X}}}}}}^{v}}p(s)\ln q(s)ds}_{{{{{{{{\rm{depends}}}}}}}}\,{{{{{{{\rm{on}}}}}}}}\,x(t)}$$

7 $$=-{\int}_{{{{{{{{{\mathcal{X}}}}}}}}}^{v}}p(s)\ln q(s)ds$$

8 $$=-{\int}_{{{{{{{{{\mathcal{X}}}}}}}}}^{v}}p(s)\ln q(s)ds$$

9 $$=-{{\mathbb{E}}}_{p(s)}\left[\ln q(s)\right],$$

where ${\mathbb{E}}$ is the expectation operator, *q*(*s*) = *q*(*s*∣*x*(*t*)), and *p*(*s*), $p(s)\ > \ 0,{\int}_{{{{{{{{{\mathcal{X}}}}}}}}}^{v}}p(s)ds=1$, is a distribution that describes where in the search domain an informative measurement is likely to be acquired. Note that we drop the first term in the expanded KL-divergence because it does not depend on the trajectory of the agent *x*(*t*).

We approximate the expectation operator using

10 $${D}_{{{{{{{{\rm{KL}}}}}}}}}(p\parallel q)=-{{\mathbb{E}}}_{p(s)}\ln q(s)$$

11 $$\approx -\mathop{\sum }\limits_{i=1}^{N}p({s}_{i})\ln q({s}_{i})$$

12 $$\approx -\mathop{\sum }\limits_{i=1}^{N}p({s}_{i})\ln \int\nolimits_{{t}_{0}}^{{t}_{f}}\exp \left[-\frac{1}{2}\parallel \left(s\right.-\bar{x}(t){\parallel }_{{{{\Sigma }}}^{-1}}^{2}\right]dt$$

where *N* is the number of samples in the search domain drawn from a uniform distribution. With this formulation, we are able to obtain the benefits of indirectly sampling from the spatial distribution *p*(*s*) without having to directly compute derivatives to generate an optimal control signal. Furthermore, this sample-based measure prevents the computation from scaling exponentially with the number of exploration states.

### KL-ergodic exploration (KL-E^2^)

We now synthesize optimal control using the derivative of the measure in Eq. (10) with respect to the duration time *λ* of control μ_*_(*t*), or the mode insertion gradient, which calculates the sensitivity of the measure with respect to duration.

The first order sensitivity of (10) with respect to the control duration *λ* of the applied control *μ*_⋆_(*τ*) is

13 $$\frac{\partial J}{\partial \lambda }{| }_{t = \tau }=\rho {(\tau )}^{\top }({f}_{2}-{f}_{1})$$

where *f*_2_ = *f*(*x*(*t*), *μ*_⋆_(*t*)) and *f*_1_ = *g*(*x*(*t*)) (or the unactuated dynamics), and $\rho (t)\in {{\mathbb{R}}}^{n}$ is the adjoint, or co-state variable is the solution to the differential equation:

14 $$\dot{\rho }=-\left(-\frac{\eta }{{T}_{r}}\mathop{\sum}\limits_{i}\frac{p({s}_{i})}{q({s}_{i})}\left(\frac{\partial \mu }{\partial x}\right)\right)-{\left(\frac{\partial f}{\partial x}\right)}^{\top }\rho .$$

Given the mode insertion gradient, we need to find the control *μ*_⋆_(*t*) that most significantly decreases (13) by writing it as an unconstrained optimization problem:

15 $${J}_{2}=\int\nolimits_{{t}_{i}}^{{t}_{i}+T}\frac{\partial }{\partial \lambda }J{| }_{t = \tau }+\frac{1}{2}\parallel \left.{\mu }_{\star }(t)\right){\parallel }_{R}^{2}$$

where $R\in {{\mathbb{R}}}^{m\times m}$ is a positive definite matrix that penalizes the deviation from the policy *μ*(*x*). The control vector that minimizes *J*_2_ is given by

16 $${\mu }_{\star }(t)=-{R}^{-1}h{(x(t))}^{\top }\rho (t).$$

Supplementary Fig. 4 shows the results of the KL-E^2^ controller exploring a target distribution. The controller generates a trajectory that ergodically explores with respect to the target distribution (i.e., it explores proportionally with respect to the target distribution). However, the Fourier-based ergodic metric, which scales exponentially ${\mathbb{O}}(n* | k{| }^{n})$, where ∣*k*∣ is the maximum-integer Fourier term and *n* is the dimensions of the exploration space. On the other hand, our proposed approach scales linearly ${\mathbb{O}}(| k| * n)$, where ∣*k*∣ is the number of samples, making it more computationally efficient for real-time, high-dimensional exploration. Given this control formulation, all that is needed is to define a target spatial distribution that specifies which measurements are more informative for estimating the sensor model.

### Importance measure for sensor model estimation

To generate a target coverage distribution for the agent, we construct a measure that allows the agent to quantify where in the search domain will be useful data to best improve its learning of the sensor model. Here, we use the CVAE network to generate an estimate of data importance over the search space.

**Definition 2** The importance measure $\nu :{{{{{{{\mathcal{X}}}}}}}}\times {{{{{{{{\mathcal{X}}}}}}}}}^{v}\to {{\mathbb{R}}}^{+}$ for a new state *s* in the search domain when the agent is at its current state *x* is given by

17 $$\nu (s,x)=\exp {\left({\mathbb{H}}({p}_{{\theta }_{d}(y| {z}_{x},s)})\right)}^{k}$$

where ${\mathbb{H}}$ is the entropy of the decoder network ${p}_{{\theta }_{d}}$ conditioned on a given robot state *s* and the latent space representation *z*_*x*_ generated from the robot’s current state *x* and *k* is an effective temperature parameter for the distribution that exponentially weighs regions of high importance.

As we assume a normal distribution, the entropy does not depend on the mean of *p*_*ϕ*_(*y*∣*z*, *x*)^11^. Since the CVAE converts the inputs into a statistical latent space, we use the estimate of the decoder network entropy as a function of state to represent model uncertainty over the workspace—generating an expected information density. We use this expected information density distribution to specify ergodic coverage, such that the robot prioritizes regions with the most informative data to best improve the latent space representation.

**Definition 3** The importance distribution $\nu \in {{\mathbb{R}}}^{+}$ is defined as the entropy of the normal distribution,

18 $$\nu (s,x)\propto \exp {\left(\frac{1}{2}\ln [{\sigma }_{y}^{2}(s,x)]\right)}^{k}$$

where points $s,x\in {{{{{{{{\mathcal{X}}}}}}}}}^{v}$ and the robot’s current state is *x*.

### Algorithm overview

We provide an outline of our KL-E^2^ method in Algorithm 1 for real-time active learning for sensor model estimation.

#### Algorithm 1

KL-Ergodic exploration (KL-E^2^) for variational autoencoders

  1: **initialize:** local dynamics model *f*(*x*, *u*), initial condition *x*(*t*_0_), CVAE network *C*, Learned parameters *θ*, *ϕ* of network *C*, importance distribution *p*(*s*, *x*), number of CVAE optimizations *j*_*opt*_, batch size *b*.

  2: **for** *i* = 0, …, *∞* **do**

  3:  Calculate optimal control *μ*_⋆_(*τ*) for $t\in \left[{t}_{i},{t}_{i}+T\right]$

  4:  Calculate application time and duration *τ*, *λ* that minimizes $\frac{\partial }{\partial \lambda }J$

  5:  Apply ${\mu }_{\star }(\tau ){{{{{{{\rm{if}}}}}}}}t\in \left[\tau ,\tau +\lambda \right]{{{{{{{\rm{elseapply}}}}}}}}\,\mu (x(t))$ to robot

  6:  Sample state *x*(*t*_*i*_) and measurement *y*(*t*_*i*_)

  7:  **for** *j* = 0, …, *j*_opt_ **do**

  8:   Generate minibatch *M* by randomly sampling *b* data points from full set

  9:   Generate random noise *ϵ* ~ *p*(*ϵ*) for each data point in *M*

10:   Compute loss *L*_*θ*,*ϕ*_ and its gradients ∇_*θ*,*ϕ*_*L*_*θ*,*ϕ*_

11:   Update *θ* and *ϕ* using SGD optimizer

12:  **end for**

13:  Generate latent space representation *z*_*x*_ at current robot state *x*_*i*_

14:  Update importance distribution *p*(*s*, *x*)

15: **end** **for**

## Supplementary information

Supplementary Information

Description of Additional Supplementary Files

Supplementary video

## Data Availability

The data for all experiments and figures in the paper are publicly available. A downloadable version of the datasets used for the different sensory simulations in this paper is archived and links for them can be found on the github reposity https://github.com/apr600/mechanical-intelligence.

## Code availability

Implementations of the active sensory learning algorithm with the different environment examples are available at https://github.com/apr600/mechanical-intelligence. This package also contains code reproducing the main results from the paper.
