# Supplementary material for: Mechanical intelligence for learning embodied sensor-object relationships
Source: Nat Commun. 2022 Jul 15;13:4108. doi: 10.1038/s41467-022-31795-2 (PMC9287329; doi:10.1038/s41467-022-31795-2)
Supplement: Supplementary file 1 — Supplementary Information [file 41467_2022_31795_MOESM1_ESM.pdf]

Mechanical Intelligence for Learning Embodied Sensor-Object Relationships: *Supplementary Information*

Ahalya Prabhakar and Todd Murphey

A Preliminaries

A.1 Variational Autoencoders

The variational autoencoder [1] is a graphical model that relates a distribution of hidden latent variable representation z to that of the data y. The autoencoder network consists of two components, an encoder-decoder neural network pairing that compresses the high-dimensional input through a bottleneck over a latent space through the encoder network and reconstructs the input from the latent variables through the decoder, ensuring that the reconstruction is only dependent on minimal features of the input (where the number of features is determined by the chosen dimension of the latent space). The *conditional* variational autoencoder (CVAE) first introduced by [2] is an extension of the VAE that models the latent variables and data, both conditioned on a random variable x. It works as follows: a set of latent variable z is generated from the prior distribution $p_{\theta_e}(z|y,x)$ and the $\tilde{y}$ is generated from the generative distribution $p_{\theta_d}(y|z,x)$, where $z \sim p_{\theta_e}(z|x,y), y \sim p_{\theta_d}(y|z,x)$.

The loss function of the CVAE consists of minimizing the reconstruction loss with a KL-Divergence measure as a regularizer. The variational lower-bound of the conditional likelihood used as the objective to be minimized is as follows:

$$\mathbb{L}(\theta_d, \theta_e; y, x) = -\mathbb{E}[\log p_{\theta_d}(y|z,x)] - D_{KL}[p_{\theta_e}(z|y,x)||p(z)]. \tag{1}$$

The variational autoencoder represents the input as a statistical distribution over the latent space—this ensures that the latent space is continuous, enabling interpolation for predicting new inputs, which is necessary when learning a generative measurement model. We employ the CVAE, where the model is explicitly conditioned on the agent state. With a CVAE, we learn a model that will predict the most likely sensor data given the state (shown in Supplementary Figure 1)—exactly the role of a measurement model in general.

B Experiment Details

B.1 Sensory Environments

B.1.1 Electrosense

Electrolocation is a near-field sensing modality found in a type of freshwater fish that relies on measuring disturbances in a self-generated, weak electric field [3, 4, 5]. The near-field nonlinear sensor emits electromagnetic fields and uses variations in voltage potentials to detect underwater conductors. Because the sensing range for electrolocation is small, the system (fish or robot) must be relatively near the object in order to localize it. Furthermore, the sensors are rigid with respect to the body, so the sensor dynamics are defined only by the relative dynamics of the body and the conductor. The electrosensory measurement model (shown in Supplementary Figures 2 and 3) measures the relative voltage differential between two excitation electrode sensors on the robot body—as described here, our simulations have 441 of these voltage differentials located across the body. This voltage differential results in a highly nonlinear response to objects in the environment generating perturbations to the self-generated electric field.

For simulation, we use a physics-based measurement model that approximates the response of the electrosensory robotic system from [3]. Similar to the electrosensory system found in electric fish, which can have 14000 electrosensory receptors distributed across its body [5], here we simulate a measurement model that outputs a 21x21 sensor measurement experienced around the body of the robot, as shown in Supplementary Figure 3. Each of the 441 ”pixels” in this signal can be considered as the result of the voltage differential of two electrodes on the body of the robot. The learning model consists of a standard conditional variational autoencoder formulation, shown in Supplementary Figure 7, which takes in a 441-dimensional measurement y and corresponding robot state $x = [p_x, p_y]$. Using this, it generates a predicted sensor signal, flattened into a 441-dimensional input data for a given robot state $[p_x, p_y]_r$. We have no a priori information about the measurement model nor about where the object is in the environment during the learning process. Because the dataset has to be acquired and we want the learning process to influence the data collection, the exploration and learning phases run concurrently, where each exploration step is followed by a learning phase. Each learning phase consists of 10 steps (also known as an minibatch gradient descent), which optimizes CVAE model parameters using randomly sampled subsets of the full dataset of a chosen batchsize (here, we choose 64-sample batchsize) [6]. Once the learning phase is complete, the entropy-based target distribution is updated to guide the active exploration. The learning phases only begin once the exploration steps have acquired enough data samples to perform the first learning phase (i.e., 64 physical steps). The exploration ran for a total of 1000 exploration steps.

To evaluate the learned model, we seed the model using a test datapoint at the center of the workspace (at $[0, 0]$) to seed the model and generate the latent space encoding. The latent space encoding is used to predict the sensor output when the robot is directly over the conductor object (at $[0.4, 0.68]$), using the mean squared error loss between the predicted sensor output and the physics-based measurement to evaluate the learned

model.

B.1.2 1-Channel Intensity Camera

We simulate a Pybullet[7, 8] environment with a Franka Panda Emika Robot with a camera on at its end effector. We set the simulated camera arm at a constant height above the table and orientation at $[0, -\frac{\pi}{2}, 0]$ such that the camera is always pointing downward (shown in Supplementary Figure 6). The camera sensor outputs 1-channel intensity 38x38 pixel images measured at the robot state. We input the flattened 1-channel 38x38 intensity image (1444-dimensional) sensor output y_r and the 2-dimensional robot state $x_r = [p_x, p_y]$ into the learning network. We use the ergodic sampling algorithm with an IK solver—which sets robot joint states to achieve a desired end-effector location—to explore the environment and acquire data for learning. The agent explores and learns in an environment containing two objects—a cube and a sphere. The sensor model consists of a conditional variational autoencoder network with an initial convolutional neural network attached, as shown in Supplementary Figure 8. The learning takes place in two stages—during exploration for 1500 physical steps to acquire data, with a learning phase consisting of 10 gradient descent minibatch learning steps between each physical step, with a randomly sampled subset of the dataset (using a batchsize of 64). An additional 1000 minibatch learning steps were performed after exploration. The learned model is evaluated on a test dataset (shown in Supplementary Figure 10) that consists of 18 data points, 9 robot states in a grid over each object, where the object is always in camera view. For each of the objects, the model is seeded with the sensor output at the robot state directly over the object to generate a sampled z from the latent space, from which the output is predicted at the 9 states close to that object. The loss is calculated as the mean squared error between the predicted outputs and the actual sensor data. Energy expenditure is calculated as the cumulative distance between each state over the trajectory. The results for this example are further discussed in the additional results (Section C).

B.1.3 3-Channel RGB Camera

We simulate a Pybullet[7] environment with a Franka Panda Emika Robot with a camera on at its end effector.We set the camera arm at a constant height above the table and orientation at $[0, -\frac{\pi}{2}, 0]$ such that the camera is always pointing downwards, (shown in Supplementary Figure 6). The camera sensor outputs 3-channel RGB 75x75 pixel images y_r at the corresponding robot state x_r. We use the ergodic sampling algorithm with an IK solver—which sets robot joint states to achieve a desired end-effector location—to explore the environment and acquire data for learning. The simulated environment contains a rubber duck on laying on its side of which the robot is exploring and learning a sensory representation. Similar to before, there is a two-stage learning process: first simultaneously with exploration for 1000 physical steps, with a learning phase consisting of 10 learning iterations between each step, and an additional 10,000 learning iterations afterwards using the full acquired dataset. The learned model is evaluated on a test dataset that consists of 9 robot states in a grid over the duck. The model is seeded with the sensor output at the robot state directly over the duck to generate

the latent space z, from which the output is predicted for each of the states. The loss is calculated as the mean squared error between the predicted outputs and the actual sensor data. Energy expenditure is calculated as the cumulative distance between each state over the trajectory. Supplementary Figure 13 shows the results of the evaluation. Using ergodic sampling results in a better quality learned model throughout learning compared to using random sampling. Furthermore, it is able to do so with significantly less energy expenditure during the exploration phase.

C Additional Results

Intensity Camera Model Learning with Franka Robot

Complex environments can often contain *multiple objects* that need to be explored to accurately encode the scene and to discriminate between the objects. We illustrate such an example with 1-channel intensity image learning for a camera attached to a Franka robot arm on a tabletop environment with two objects of different shapes and textures in it (shown in Supplementary Figure 9). We compare the results from exploring with random sampling as the benchmark as well as two active exploration algorithms based on entropy—an information-maximization algorithm and the proposed ergodic sampling algorithm. We generate a learned model using an sampling-based information maximization algorithm, similar to [9, 10], that seeks the maximum of the entropy distribution by sampling near its current state and moving to the location of maximum entropy. We compare this to the proposed entropy-based active exploration algorithm using ergodic sampling. The random sampling generates samples uniformly over the entire workspace, while the information-maximization algorithm spends more time exploring near one region of high information (i.e., near one of the objects in the environment). In contrast, the ergodic sampling strategy spends more time acquiring data where the model is most uncertain (i.e., near the two objects in the environment). Importantly, the proposed ergodic sampling algorithm results in exploration around *both* objects of interest without getting stuck in local minima (i.e., only exploring near one of the objects) as it explores *proportionally* to the target distribution instead of following the gradient ascent to a peak.

Supplementary Figure 9 compares the results of the learned visual models for a data point from the test data set near the objects in the environment. Using the robot state and data directly over the object to generate a sampled z from the latent space, we predict the resulting camera image at robot location near the object to compare the conditional representation of the camera model. While the random sampling model is able to correctly predict the object's location in the image, the learned model using the ergodicity-based exploration generates a better reconstruction of the objects in the camera image. The ergodicity-based model captures the shape and texture characteristics of the different objects, while random sampling model is not able to capture and discriminate the different shapes (i.e., the square and circular features of the cube and ball). An important consequence is that the model learned from random sampling would not be able to *discriminate* between the sphere and the ball, whereas the model learned from active exploration can. The entropy-based information-maximization al-

gorithm results in an accurate reconstruction of the sphere (which it spends most of the time exploring), but also results in significantly worse performance over the cube— it does not capture the cube's location with relation to the camera.

Supplementary Figure 10 shows the comparison of the learning loss over the learning process and energy expenditure during data acquisition between ergodic, information-maximizing and random sampling. Ergodic sampling results in better quality learned sensory model, capable of predicting a more accurate sensor output experienced at novel states compared to random sampling and information maximization. Furthermore, because it explores proportional to the entropy-based information distribution, it is able to do with less energy expenditure during exploration, while still capturing the features of both objects in the environment.

Overall, the benefits of active, informative data acquisition are critical for scenarios with complex sensors and environments where learning is computationally expensive and require large amounts of data that can be energetically expensive to acquire.

C.0.1 Ablation Studies

We conduct an ablation study on the proposed algorithm to investigate the effect of hyperparameter choices on exploration and resulting learning performance. Supplementary Figure 11 shows the effects of the target distribution sample size on algorithm performance in the multi-object environment with the grayscale camera. We test the performance of the ergodic sampling algorithm using 10, 50, 100, 200, and 500 samples of the target distribution each step. We see that as the number of target distribution samples increases, the resulting exploration strategy is more concentrated over the regions of the objects— though they do not simply remain directly above the object. As a result, the performance of the learned model improves with increasing sample size, but there are diminishing returns as the sample size increases. We also observe the effect of choice of time horizon during optimization on resulting performance. Supplementary Figure 12 shows the results of testing different time horizons (i.e., number of steps into the future during optimization) in the multi-object environment with the grayscale camera. We test the performance using a horizon of 5, 10, 20, and 50 steps. We see that as the time horizon increases, the performance worsens. This is because with long planning time horizons, we assume that the desired target distribution has fewer learning updates. This assumes more time to optimize with respect to the desired distribution and can result in more conservative control actions at each time step. As such, the resulting data acquisition does not explore the workspace sufficiently and results in degraded learning.

D Further Discussion

While the problem of exploration for learning could be posed as a Markov Decision Process (MDP) in a reinforcement learning (RL) framework, the goal and problem framework of the proposed algorithm is different from standard RL problem formulations, as shown in Supplementary Figure 14. The sensory learning problem is an unsupervised learning problem where the goal is to learn the sensor-object relation-

ships from acquired unlabelled data. In reinforcement learning the goal is to learn an optimal policy to maximize a given reward function. In this work, the learning process seeks to learn the desired sensor model, not an optimal policy. The optimal control introduced here affects the *data acquisition process* that guides the learning in order to maximize the efficiency of the model learning. Furthermore, reinforcement learning require many rollouts of exploration in order to learn an optimal policy that, given a state, generates the action that maximizes the reward function.

While recent work has investigated reinforcement learning that rewards curiosity by generating a reward on data that is not predicted [11], these approaches require many rollouts to learn an optimal policy. In contrast, this work develops a model-predictive controller that actively explores the space with respect to the learned network structure and the entropy of the learned latent space. In this setting, there are no rollouts or multiple attempts needed to generate optimal behavior. Instead, we define a model predictive control (MPC) framework that explores the environment and actively acquires the most informative data to aid the sensory learning process in a single rollout. Moreover, the MPC process is only needed if the dynamical system does not achieve ergodic coverage on its own. Some systems may be ergodic—and therefore learn with the benefits of ergodic coverage discussed in this paper—as a result of their mechanics and without requiring any computational synthesis.

Supplementary References

[1] Kingma, D. P. & Welling, M. Auto-encoding variational Bayes. In *Proceedings of the International Conference on Learning Representations (ICLR)* (2014).

[2] Sohn, K., Lee, H. & Yan, X. Learning structured output representation using deep conditional generative models. In Cortes, C., Lawrence, N. D., Lee, D. D., Sugiyama, M. & Garnett, R. (eds.) *Advances in Neural Information Processing Systems 28*, 3483–3491 (2015).

[3] Miller, L. M., Silverman, Y., MacIver, M. A. & Murphey, T. D. Ergodic exploration of distributed information. *IEEE Transactions on Robotics* **32**, 36–52 (2016).

[4] Krahe, R. & Fortune, E. S. Electric fishes: neural systems, behaviour and evolution. *Journal of Experimental Biology* **216**, 2363–2364 (2013).

[5] Nelson, M. E., MacIver, M. A. & Coombs, S. Modeling electrosensory and mechanosensory images during the predatory behavior of weakly electric fish. *Brain, Behavior and Evolution* **59**, 199–210 (2002).

[6] Goodfellow, I., Bengio, Y. & Courville, A. *Deep Learning* (MIT Press, 2016).

[7] Coumans, E. & Bai, Y. Pybullet, a python module for physics simulation for games, robotics and machine learning. `http://pybullet.org` (2016–2021).

[8] Busy, M. & Caniot, M. qibullet, a bullet-based simulator for the pepper and nao robots. *arXiv preprint arXiv:1909.00779* (2019).

[9] Bourgault, F., Makarenko, A. A., Williams, S. B., Grocholsky, B. & Durrant-Whyte, H. F. Information based adaptive robotic exploration. In *IEEE International Conference on Intelligent Robots and Systems*, 540–545 (2002).

[10] Vergassola, M., Villermaux, E. & Shraiman, B. I. 'Infotaxis' as a strategy for searching without gradients. *Nature* **445**, 406–409 (2007).

[11] Pathak, D., Agrawal, P., Efros, A. A. & Darrell, T. Curiosity-driven exploration by self-supervised prediction. In *International Conference on Machine Learning*, 2778–2787 (2017).

E Supplementary Figures

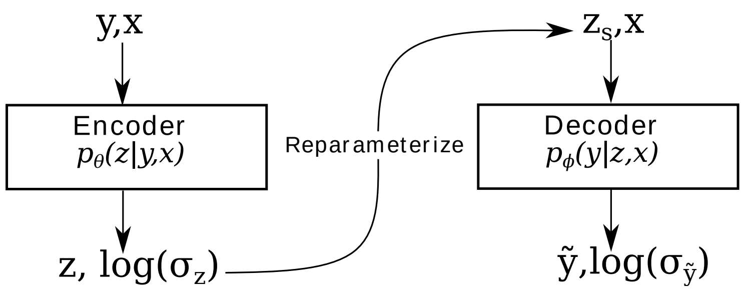

Supplementary Figure 1: **Conditional Variational Autoencoder.** The conditional variational autoencoder consists of a encoder-decoder neural network pairing for high-dimensional data compression. Here, the encoder takes in input sensor data y and the conditional state (the robot state) x and encodes it into a latent representation space z with log variance $\log(\sigma(z))$. The decoder network takes in samples z_s and the conditional state (the robot state) x and outputs the reconstructed sensor data $\tilde{y}$ and its log variance $\log[\sigma(\tilde{y})]$.

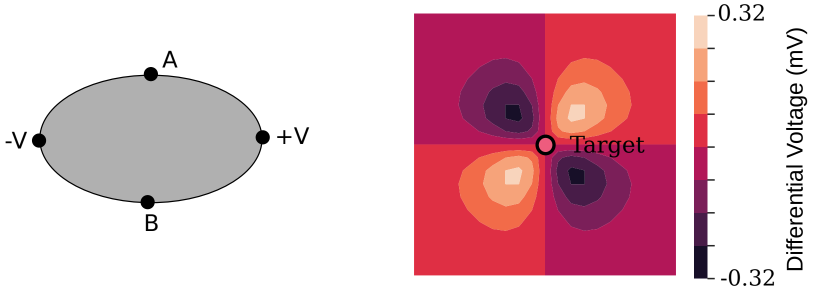

a) b)

Supplementary Figure 2: **Electrosensory Model for a Single Electrode.** (a) In the electrosensory modality, an electric field is generated by two electrodes on the body marked with a positive and negative voltages at each end of the robot. The difference between the field voltage at the two sensors (marked as A,B) on the robot body. Objects in the environment result in a perturbation of the electric field and a resulting voltage difference experienced from what is expected. This voltage differential is relative to the position of the object with respect to the robot. (b) The physics-based measurement model simulation approximates the response of the electrosense robotic system from [3]. The function captures the voltage differential experienced by the robot between pairs of sensors on the robot body.

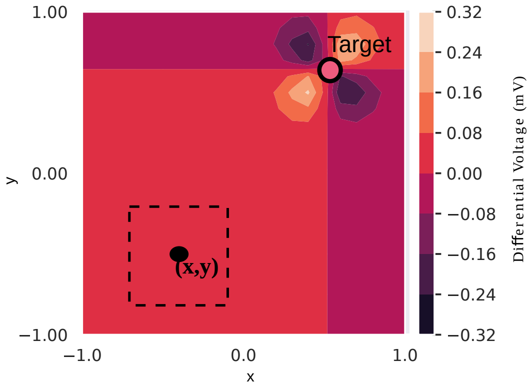

Supplementary Figure 3: **Electrosensory Model.** The physics-based measurement model simulation approximates the response of the electrosense robotic system from [3], shown here responding to a spherical object (marked with a pink target) located at approximately [0.4, 0.68]. The measurement model outputs a 21x21 evenly-spaced grid array signal covering the region (indicated by the dotted region with dashed black outline) around the robot.

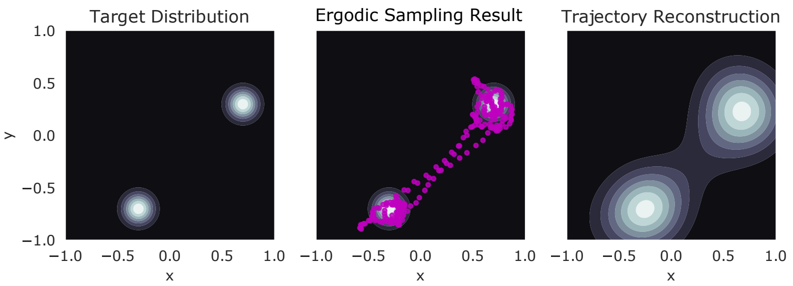

Supplementary Figure 4: **Result of Ergodic Sampling Controller.** Exploring the target distribution (right), the ergodic sampling algorithm actively explores the search space, generating a trajectory (middle) proportional to the target distribution. The time-averaged distribution reconstruction of the resulting trajectory matches the statistics of the target distribution.

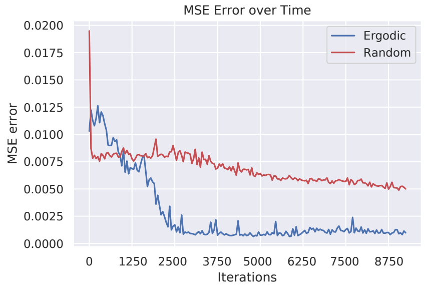

Supplementary Figure 5: **Electrosense Testing Loss Comparison with Random Sampling and Active Ergodic Sampling.** We show the testing loss for the electrosensory measurement field estimates directly over the object for each method. MSE loss is significantly lower for the ergodic sampling (shown in blue) compared to random sampling (shown in red).

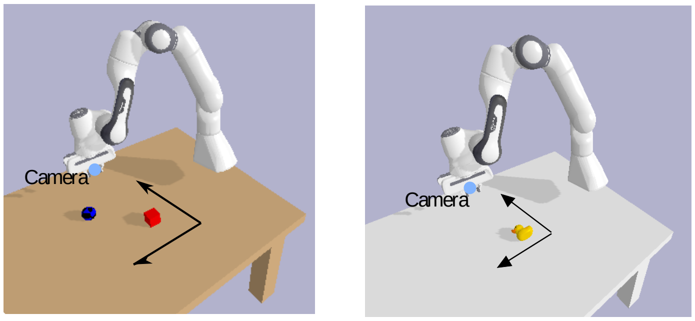

Supplementary Figure 6: **Simulator Environments for the Intensity and RGB Camera.** The Pybullet environments contain a Franka Panda Emika Robot, with base located at [0,0,0]. The camera (indicated with a blue dot) is attached at the end effector of the robot, at a height of $y = 0.3$, pointing directly downwards at the table. The environment workspace has a exploration space of $[[-0.2, 0.2], [-0.3, -0.7]]$. For the RGB camera environment, the duck is located at [-.1, -0.4]. For the intensity camera environment, the cube is located at [-1.,-0.4] and the sphere is located at [0.1, -0.6].

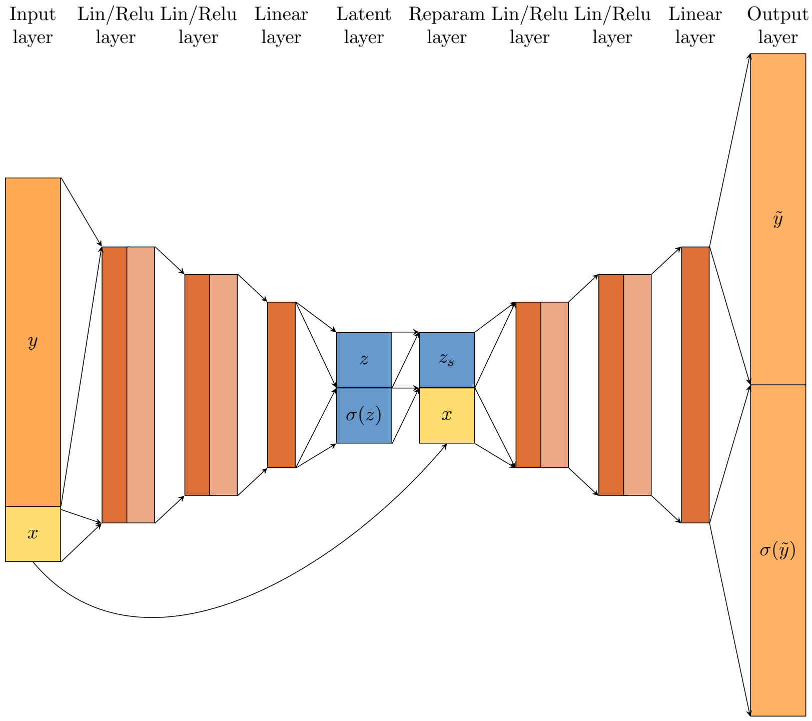

Supplementary Figure 7: **CVAE Network Setup used for Learning Electrosense Model.** The model consists of a standard conditional variantional autoencoder setup. It takes in the electrosensory measurement and robot state and encodes it into a latent space. This latent space is then used to reconstruct the measurement (possibly at a different robot state).

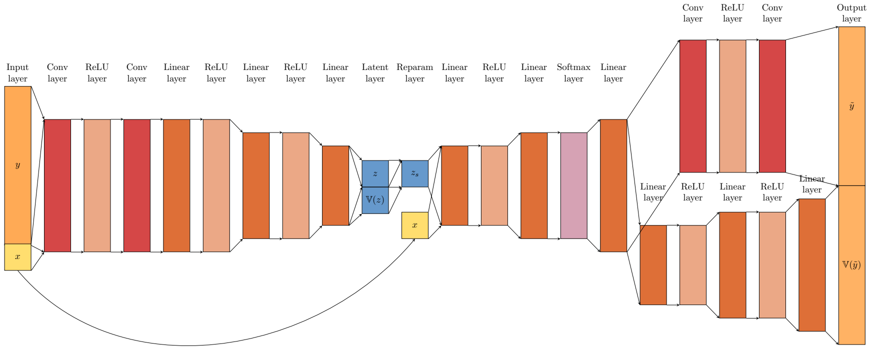

Supplementary Figure 8: **CVAE Network Setup for Camera-based Models.** The model consists of a conditional variantional autoencoder formulation with a convolutional neural network for processing the high-dimensional images from the camera.

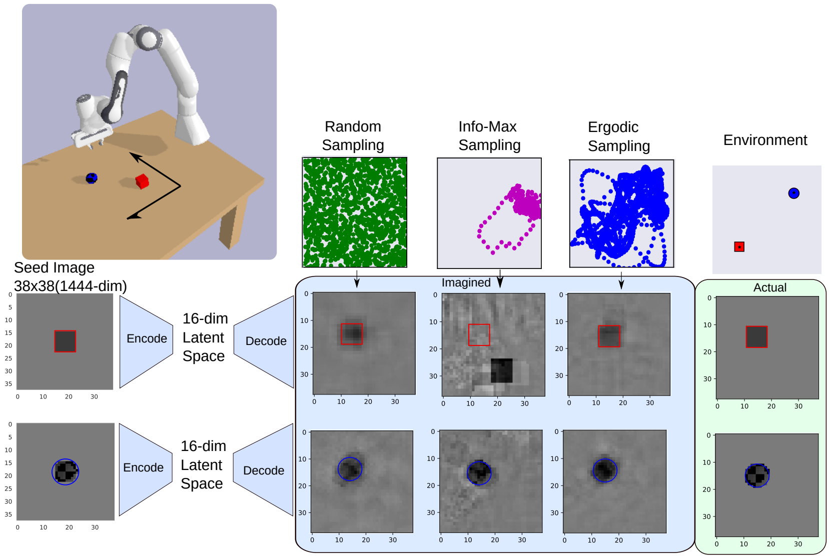

Supplementary Figure 9: **Intensity Camera Model Learning.** Here, we compare the predicted measurement generated from the learned intensity camera model for two different objects in the environment. We compare the results for random sampling, entropy-based information-maximizing algorithm and entropy-based ergodic sampling. While the entropy-based information-maximization algorithm recognizes the location of an object of interest in the environment, the method focuses on one region of interest (i.e. object) to explore. The ergodic sampling method, on the other hand, spends time exploring near *both* objects in the environment (indicated with black markers), resulting in learning around the multiple regions of interest within the environment, with no prior information given about the number or locations of objects in the environment. Using the seed images over the object location to generate the latent space representation, we compare the predictions of the camera images from near the objects, using the learned models from random sampling, entropy-based information-maximization and the proposed active learning approach. While both learned models capture the object location, the entropy-based ergodic sampling approach results in a better reconstruction of the objects, capturing the distinctive features of the two different objects (i.e., shape of the objects), such that the predictions can discriminate between the two different objects. The predicted reconstructions generated from the model learned with random sampling, on the other hand, is does not reflect the individual objects' characteristics such that object discrimination could be accomplished.The entropy-based information maximization approach results in a learned model that accurately reconstructs the object it explores, but does not capture the other object's features or location.

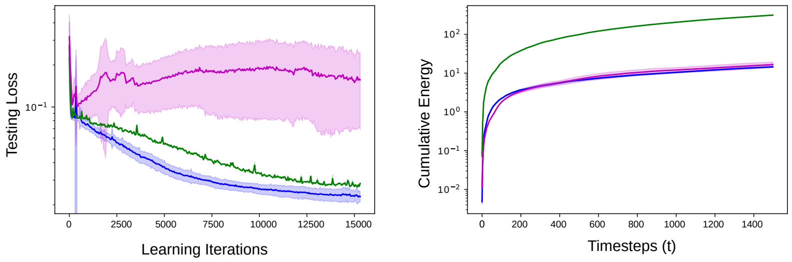

Supplementary Figure 10: **Comparison of Testing Loss for Intensity Camera Estimate and Energy Expenditure using Random Sampling, Information Maximization and Ergodic Sampling.**(a) Mean Testing Loss for the intensity image estimates for the test data set (consisting of points near the objects of interest) for each method from 10 random initial states. The shaded area shows the standard deviations for the testing loss over the 10 trials. Loss is significantly lower for the ergodic sampling(shown in blue) compared to random sampling (shown in green) or information maximization (shown in magenta). (b) Cumulative Energy over time for the trajectories from the exploration strategies. The energy used for data acquisition from ergodic sampling is significantly lower compared to random sampling (shown in green). While the energy expenditure for the information maximization approach is similar to that of ergodic sampling, it results in a significantly worse performance (higher loss) compared to ergodic sampling.

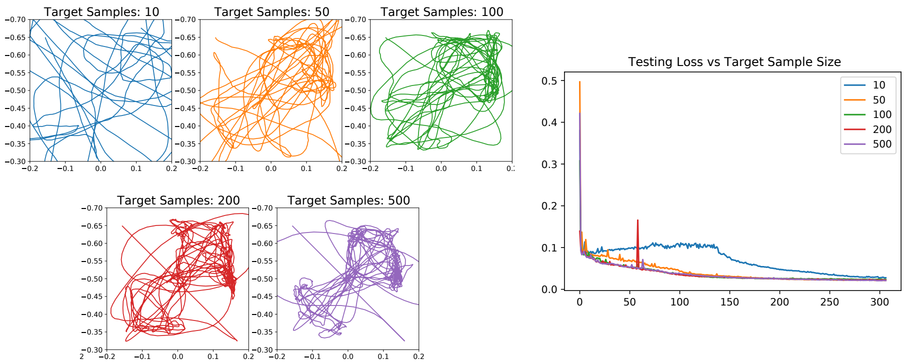

Supplementary Figure 11: **Effect of Target Distribution Sample Size on Exploration Trajectory using Ergodic Sampling.** We show the resulting exploration trajectories and testing losses using different sample sizes of the target entropy distribution using the proposed approach. As the sample size increases, the exploration becomes more concentrated in the regions around the two objects. The trajectory spends more time in the regions of high information, resulting in a lower testing loss with higher sample size.

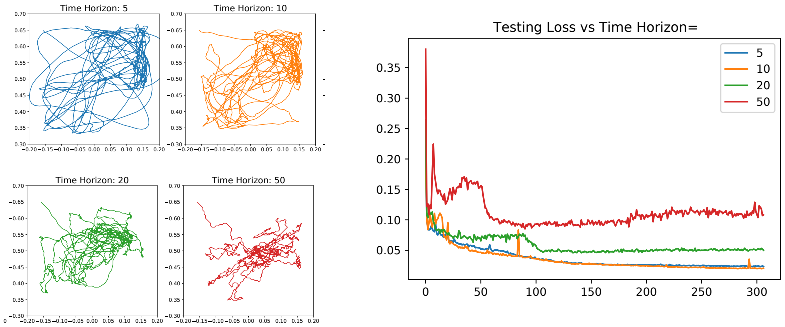

Supplementary Figure 12: **Effect of Time Horizon during Ergodic Sampling Algorithm on Exploration Trajectory and Learning Performance.** We show the exploration trajectory and resulting testing losses from learned models using different time horizons for optimization in the proposed approach. As time horizon increases,the exploration performance degrades. This is due to the fact that with a longer time horizon, we assume fewer updates of the target distribution and therefore more time to optimize with respect to the target distribution. This results in more conservative control actions at each time step and less exploration over the workspace, resulting in worse learning performance.

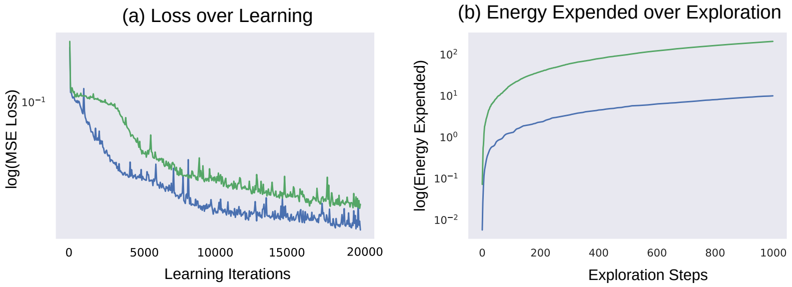

Supplementary Figure 13: **Comparison of Testing Loss of RGB Measurement Estimate and Energy Expenditure using Random Sampling and Ergodic Sampling.** (a) Testing Loss for the RGB image estimates for the test data set (consisting of points near the rubber duck in the environment) for each method. Testing loss is significantly lower for ergodic sampling (shown in blue) compared to random sampling (shown in green). (b) Cumulative Energy over time for the trajectories from the exploration strategies. The energy used for data acquisition from ergodic sampling is significantly lower compared to random sampling (shown in green).

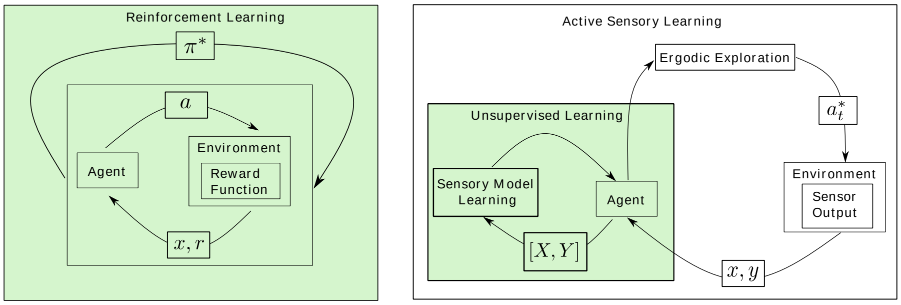

Supplementary Figure 14: **Comparison of Reinforcement Learning Problem Overview and Sensory Learning Problem Overview.** The goal of reinforcement learning is to learn the optimal policy for a task by rolling out many trials and iterating over them to learn a model that represents the optimal policy for the task. In this work, we focus on learning a sensory model using unsupervised learning. The ergodic sampling algorithm is used to actively explore and gather data for the learning process. The ergodic control generates the optimal action for active learning, which only reflects the utility of the data gathered.
