# Supplementary material for: Mechanical intelligence for learning embodied sensor-object relationships
Source: Nat Commun. 2022 Jul 15;13:4108. doi: 10.1038/s41467-022-31795-2 (PMC9287329; doi:10.1038/s41467-022-31795-2)
Supplement: Supplementary file 2 — Description of Additional Supplementary Files [file 41467_2022_31795_MOESM2_ESM.pdf]

Description of Additional Supplementary Information

Supplementary Video: Supplementary movie with overview of method and videos of examples from the work.
